# Enhancing ICU *Candida* spp. surveillance: a cost-effective approach focused on *Candida auris* detection

**DOI:** 10.3389/fcimb.2024.1463456

**Published:** 2024-11-01

**Authors:** Teresa Nascimento, João Inácio, Daniela Guerreiro, Priscila Diaz, Patrícia Patrício, Luís Proença, Cristina Toscano, Helena Barroso

**Affiliations:** ^1^ Microbiology, Egas Moniz Center for Interdisciplinary Research (CiiEM), Egas Moniz School of Health & Science, Almada, Portugal; ^2^ School of Applied Sciences, University of Brighton, Brighton, United Kingdom; ^3^ Intensive Care Unit, Hospital Prof. Doutor Fernando da Fonseca, Amadora, Portugal; ^4^ Intensive Care Unit, Hospital Beatriz Ângelo, Loures, Portugal; ^5^ Clinical Pathology, Centro Hospitalar Lisboa Ocidental Hospital Egas Moniz, Lisboa, Portugal

**Keywords:** *Candida* spp., *Candida auris*, intensive care unit, surveillance, colonization, mannitol salt agar auris, prevalence

## Abstract

**Introduction:**

*Candida auris* is an emerging pathogen that represents a worldwide health problem due to its global expansion, multidrug resistance, and difficult laboratory identification. Among the risk factors for colonization/infection by *C. auris*, a stay in an intensive care unit (ICU) stands out. This prospective multicenter study aimed to monitor the trend of the local epidemiology of *Candida* spp. and unveil the prevalence of *C. auris*.

**Methods:**

From 2020 to 2022, axillar/inguinal swabs were collected from adult patients at three points: upon admission (D1) and on the fifth (D5) and eighth (D8) days of their ICU stay. We employed culture-based screening methods combined with molecular techniques to identify *Candida* spp. down to the species level. Specific screening for *Candida auris* was conducted using a real-time PCR assay in combination with an improved selective culture medium, mannitol salt agar auris (MSAA). To validate the effectiveness of MSAA, a collection of reference *C. auris* strains representing the four major geographical clades was used.

**Results:**

We enrolled 675 patients, and 355 *Candida* isolates were retrieved from the 988 swab samples collected. From those, 185/355 (52.1%) were identified as *C. albicans* and 170/355 (47.9%) as non-*albicans Candida* (NAC). MSAA medium showed a specificity of 94.8%, albeit *C. auris* was not detected in this cohort. The dynamics of *Candida* spp. colonization by ICU were significant at the three collection points. Upon admission, *C. albicans* was associated with the Beatriz Ângelo Hospital ICU (*p*=0.003) and *C. tropicalis* with the general Hospital Professor Doutor Fernando Fonseca (FFH) ICU (*p*=0.006). *C. parapsilosis* and *C. lusitaniae* were associated with FFH ICUs, with the general ICU at D5 (*p*=0.047) and surgical ICU at D8 (*p*=0.012). The dynamics of NAC colonization by ICU were significantly different at D1 (*p*=0.011), D5 (*p*=0.047), and D8 (*p*=0.012).

**Conclusion:**

We developed and implemented a screening protocol for *C. auris* while uncovering the colonization patterns of *Candida* in the ICU. Our findings contribute to the optimization of overall patient management, ensuring that ICU protocols are resilient and adaptive to emerging fungal threats.

## 1 Introduction

In the intensive care unit (ICU), patients are at heightened risk for healthcare-associated infections due to factors such as invasive procedures, weakened immune systems, and prolonged hospital stays (Thomas-Rüddel et al., 2022). *Candida* species, including the emerging multidrug-resistant *Candida auris*, are prominent pathogens that contribute to these infections through skin colonization (Soriano et al., 2023; Lass-Flörl et al., 2024).

Despite the predominant feature of colonization, *C. auris* candidemia usually follows colonization and presents mortality rates up to 70%, resulting in this species, together with *C. albicans*, being listed on the World Health Organization (WHO) critical priority group of fungal pathogens (WHO, 2022). *Candida* species are the third leading cause of nosocomial bloodstream infections and reducing their transmission is an important aspect of ICU care (Poissy et al., 2022).

Given the critical importance of accurately screening and identifying *Candida* strains, systematic surveillance in high-risk settings, particularly among ICU patients, is essential (Ahmad and Asadzadeh, 2023). Global surveillance efforts have been implemented to monitor the spread and characteristics of *C. auris* across all continents (Worth et al., 2020; Contreras and Morgan, 2022; Taori et al., 2022; Piatti et al., 2022; de Melo et al., 2023; Rowlands et al., 2023; Vinayagamoorthy et al., 2022). However, the lack of *C. auris* surveillance in countries with low prevalence presents significant challenges to global public health, as undetected introductions and potential outbreaks could occur, leading to delayed responses and increased transmission. In this context, several studies highlight the importance of screening high-risk patients upon ICU admission, particularly those with a history of healthcare exposure in foreign hospitals (Worth et al., 2020; Briano et al., 2022; Ahmad and Asadzadeh, 2023). The early identification of potential community-acquired cases is crucial in preventing *C. auris* colonization and minimizing the risk of subsequent infections (Lass-Flörl et al., 2024).

Current methods such as polymerase chain reaction (PCR), matrix-assisted laser desorption/ionization-time of flight mass spectrometry (MALDI-TOF MS), and DNA sequencing are not universally accessible, require specialized expertise, and are time-intensive (Keighley et al., 2021; Maldonado et al., 2018). *C. auris* poses significant laboratory challenges due to frequent misidentification by standard microbiology assays and automated systems (Jones et al., 2024; de Cássia Orlandi Sardi et al., 2018). Real-time PCR and DNA sequencing are typically required for reliable identification, with the sequencing of specific DNA regions like the D1/D2 of 28S rDNA or internal transcribed spacer (ITS) helping to differentiate *C. auris* clades (Lockhart et al., 2022). Given these challenges, it is crucial to encourage and support the establishment of *C. auris* surveillance programs in a cost-effective manner. This involves providing affordable diagnostic tools to ensure that emerging cases are detected and managed swiftly. To address this gap, we aimed to assess the pattern of *Candida* colonization in ICU patients and unveil the prevalence of *C. auris*.

## 2 Methods

### 2.1 Study design

This prospective multicenter study was conducted from January 2020 to December 2022, in suburban Lisbon, at two large tertiary hospitals belonging to the Portuguese National Public Health Service: *Hospital Professor Doutor Fernando Fonseca* (FFH), an 802-bed hospital with two ICUs (general and surgery), and *Beatriz Ângelo Hospital* (BAH), a 424-bed hospital with one general ICU. Participation in the study was voluntary and authorized by signing an informed consent form. All patients under the age of 18, pregnant women, and mentally disabled individuals were not included in the study. Collection was made in the context of a daily chlorhexidine gluconate (CHG) 2% and 4% (v/v) bathing routine infection control practice. All patients had at least one CHG bath prior to swab collection, with a daily bath for the first 5 days of stay.

The sampling from each patient was performed using a non-invasive bilateral axillary/inguinal combine swab. Collection was made upon the admission of patients to the ICU (D1) and continued during the ICU stay, one the fifth day (D5) and eighth day (D8), when applicable.

Swabs were collected in a 1 ml ∑-Transwab^®^ system transport (Sigma Transwab-Liquid Amies) and processed within 48 h, using mycological cultural and molecular-based methods simultaneously.

### 2.2 The *C. auris* surveillance protocol with culture-based methods

**Figure 1:**
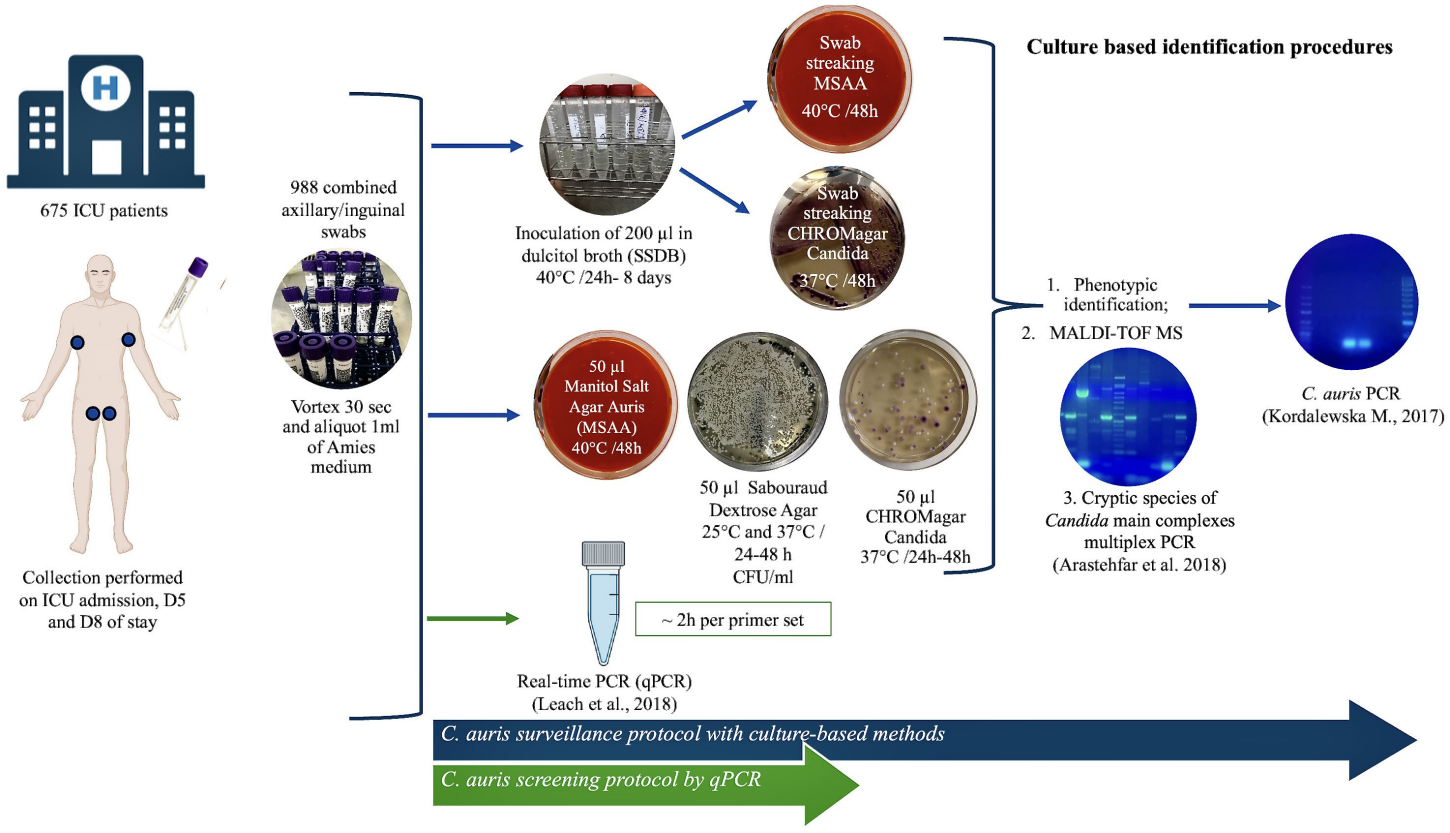
Scheme of the mycological algorithm used to screen *Candida auris*. The algorithm for the molecular identification of *C. auris* from clinical isolates (blue arrow). *C. auris* screening from surveillance samples using a specific real-time PCR (green arrow).

Briefly, for cultural methods, 50 µl aliquots of the suspensions were spread directly onto appropriate culture media: Sabouraud Gentamicin Chloramphenicol 2 agar (SDA) (bioMérieux, Marcy l’Etoile, France) and a commercially available *Candida* chromogenic medium (CHROMagar ™ *Candida*, CHROMagar, Paris, France). The plates were incubated aerobically for 48 h, one set of plates at 25°C and a second set at 37°C (**Figure 1**).

#### 2.2.1 Species identification

The presumptive identification of isolates was based on standard criteria of macroscopic and microscopic morphology, growth temperature, the biochemical profile of aerobic sugar assimilation, and the appearance on chromogenic agar (**Figure 1**). All isolates were further processed for analysis with MALDI-TOF MS—VITEK MS (bioMérieux, Marcy l’Etoile, France) using VITEK MS v3.2 software (Sarvestani et al., 2022). All identifications displaying a single result with a confidence value of 99.9% were considered acceptable (**Figure 1**).

Molecular methods were employed to analyze the three *Candida* species complexes (*C. albicans*, *C. glabrata*, and *C. parapsilosis*) most linked to human infections. The protocol, optimized from the one developed by Arastehfar et al., was designed to simultaneously identify cryptic species within the *C. albicans*, *C. glabrata*, and *C. parapsilosis* complexes (Arastehfar et al., 2018). Additionally, all *Candida* isolates were subjected to a PCR assay specific for *C. auris* (Kordalewska et al., 2017). For this purpose, total DNA was extracted from the isolates using an NZYMicrobial gDNA Isolation Kit^®^ (Nzytech, Lisboa, Portugal), following the manufacturer’s instructions. The PCRs were performed using a T100 thermal cycler (Bio-Rad Laboratories, Hercules, CA, USA). The amplified products were analyzed on 2% agarose gels stained with GreenSafe (NZYtech, Lisboa, Portugal) and visualized using a UV transilluminator (**Figure 1**).

#### 2.2.2 *C. auris* screening with mannitol salt agar auris

Aliquots of 50 μl of the swab suspensions were spread directly on an in-house selective medium for *C. auris* (MSAA) (**Figure 1**) adapted from the enrichment broth developed by Welsh et al (Welsh et al., 2017). The base of the MSAA medium was SDA supplemented with gentamicin and chloramphenicol to a 50 mg/l final concentration, to which 10% NaCl (wt./vol), 2% mannitol (wt./vol), and phenol red indicator solution, adjusted to pH 5.9, were added (Nascimento et al., 2021). A positive test result was defined as the presence of visible colony growth (yellow colonies) after 24–48 h of incubation at 40°C. A *C. auris* DSM 105987 suspension was used as positive control, prepared from a 48 h SDA culture to a concentration of 1.0 McFarland. Additionally, 200-μl aliquots of the swab suspensions were inoculated into Salt Sabouraud Dulcitol Broth (SSDB) containing dulcitol, supplemented with chloramphenicol, gentamycin, and 10% of sodium chloride for 24 h to 8 days at 40°C with shaking at 250 rpm and observed daily for turbidity. In this study, the enrichment broth was reproduced based on the description published by the Centers for Disease Control and Prevention (CDC, 2022). All the SSDB tubes after the maximum incubation time were re-inoculated (swab streaked) onto CHROMagar Candida^®^ and MSAA for 48 h at 37°C and 40°C, respectively. The results were recorded considering any colony growth on the plates and colony color (**Figure 1**).

##### 2.2.2.1 Validation of MSAA medium

A panel of *C. auris* strains representative of the four major geographical clades were obtained from the Deutsche Sammlung von Mikroorganismen (DSMZ; Braunschweig, Germany): *C. auris* DSMZ 21987, representative of South Asia Clade (I); DSMZ 105986, for the East Asia Clade (II); DSMZ 105988, for the South Africa Clade (III); and DSMZ 105990, for the South America Clade (IV). Other yeasts used to validate the MSAA medium were *Candida albicans* ATCC 60193, *Candida tropicalis* ATCC 1369, *Nakaseomyces glabrata* (formerly known as *Candida glabrata)* ATCC 15126, *Candida parapsilosis* ATCC 22019, *Pichia kudriavzevii* (formerly known as *Candida krusei*) ATCC 6258, *Candida haemulonii* DSMZ 70624, *Candida duobushaemulonii* CBS 7798, *Cutaneotrichosporon mucoides* (*Trichosporon mucoides*) ATCC 204094, and clinical isolates of *Clavispora lusitaniae* (formerly known as *Candida lusitaniae*) and *Saccharomyces cerevisiae*. All cultures were streaked onto MSAA medium using as inoculum yeast suspensions in 0.85% (v/v) saline solution adjusted to 1–5 × 10^6^ cells/ml (OD530nm: 0.128; 0.5 McFarland standard). The MSAA medium was also inoculated with all the surveillance swab sample suspensions, with and without pre-enrichment in SSDB (**Figure 1**), to evaluate its specificity to differentiate *C. auris* from other species often found as commensal organisms on the skin.

The sensitivity of the MSAA medium to detect *C. auris* at low concentration levels was assessed using serial dilutions of all the *C. auris* reference strains and other culture collection yeast strains listed above. Yeast suspensions adjusted to 1–5 × 10^5^ cells/mL (OD530nm: 0.020; 0.1 McFarland standard) were prepared and spotted in a grid fashion onto MSAA medium. The plates were incubated for 48 h at 40°C, and the appearance of the colonies was recorded after 24 and 48 h. All suspensions were also spotted onto SDA plates, which were incubated for 48 h at 30°C, as a positive control for organism viability.

### 2.3 *C. auris* screening by qPCR

In addition to the culture-based workflow for *C. auris* screening, 100-µl aliquots of the vortexed swab samples were processed with a highly accurate real-time PCR (qPCR) protocol as described previously by Leach et al. (**Figure 1**) (Leach et al., 2018). Briefly, genomic DNA was extracted from bilateral axilla and inguinal swab suspensions using an NZY Soil gDNA Isolation kit^®^ (Nzytech, Lisbon, Portugal) according to the manufacturer’s instructions. Each PCR run on the Qiagen Rotor-Gene Q included a positive extraction (*C. auris* DSM105986; 10^3^ CFU/50 μl) and positive amplification (*C. auris* DSM105990; 0.02 pg/l) controls, as well as negative extraction (reagents only) and negative amplification (sterilized nuclease-free water) controls.

### 2.4 Statistical analysis

Data analysis was carried out through descriptive and inferential methodologies using the IBM SPSS Statistics v. 29.0 (IBM Corp., Armonk, NY, USA) software. A *p*-value < 0.05 was considered statistically significant for all the above inferential analyses.

## 3 Results

### 3.1 Surveillance samples

A total of 675 patients were enrolled in the study: in 2020, 64 and 71 patients, respectively, from the general and surgical FFH ICU, and in 2021–2022, 540 patients from the BAH ICU. For 203 and 110 patients, samples were collected, respectively, within 5 (D5) and 8 (D8) days after admission to the ICU. Overall, 988 swab samples were collected from the cohort: 217 in 2020, 231 in 2021, and 532 in 2022.

### 3.2 *Candida auris* screening and the identification of isolates

*C. auris* screening by qPCR assay and culture-based methods was negative for the 988 samples. However, 371 yeast isolates were obtained from 329 culture positive samples, of which 355 corresponded to *Candida* species.

The overall results showed that *Candida albicans* remains the most frequently isolated species, representing 185 out of 355 isolates (52.1%), indicating a nearly equal distribution between *C. albicans* and non-*albicans Candida* (NAC) species. Among the NAC species, *C. parapsilosis* complex ranked second (30.7%), followed by *C. glabrata* in third (10.1%) (**Table 1**).

**Table 1:** *Candida* spp. isolates distribution by collection day and ICU^1^.

*Candida* spp. *n*=355	Total	General FFH ICU *n*=55	Surgical FFH ICU *n*=46	General BAH ICU *n*=254	*p*
*C. albicans* ss	185/355 (52.1)	18/55 (32.7)	22/46 (47.8)	145/254 (57.1)	0.004
D1	95/196 (48.5)	5/27 (18.5)	9/20 (45.0)	81/149 (54.4)	0.003
D5	53/96 (55.2)	8/19 (42.1)	7/14 (50.0)	38/63 (60.3)	0.343
D8	37/63 (58.7)	5/9 (55.6)	6/12 (50.0)	26/42 (61.9)	0.745
*C. parapsilosis* ss	109/355 (30.7)	26/55 (47.3)	15/46 (32.6)	68/254 (26.8)	0.011
D1	71/196 (36.2)	14/27 (51.9)	9/20 (45.0)	48/149 (32.2)	0.102
D5	25/96 (26.0)	9/19 (47.4)	4/14 (28.6)	12/63 (19.0)	0.047
D8	13/63 (20.6)	3/9 (33.3)	2/12 (16.7)	8/42 (19.0)	0.587
*C. glabrata* ss	36/355 (10.1)	2 (3.6)	6 (13.0)	28 (11.0)	0.202
D1	13/196 (6.6)	1/27 (3.7)	1/20 (5.0)	11/149 (7.4)	0.743
D5	14/96 (14.6)	1/19 (5.3)	3/14 (21.4)	10/63 (15.9)	0.380
D8	9/63 (14.3)	0/9 (0.0)	2/12 (16.7)	7/42 (16.7)	0.417
*C. tropicalis*	15/355 (4.2)	5/55 (9.1)	0/46 (0.0)	10/254 (3.9)	0.071
D1	11/184 (6.0)	5/27 (18.5)	0/20 (0.0)	6/149 (4.0)	0.006
D5	3/96 (3.1)	0/19 (0.0)	0/14 (0.0)	3/63 (4.8)	0.444
D8	1/63 (1.6)	0/9 (0.0)	0/12 (0.0)	1/42 (2.4)	0.776
*C. lusitaniae*	4/355 (1.1)	1/55 (1.8)	2/46 (4.3)	1/355 (0.4)	0.057
D1	2/196 (1.0)	1/27 (3.7)	0/20 (0.0)	1/149 (0.7)	0.315
D5	0/96 (0.0)	0/19 (0.0)	0/14 (0.0)	0/63 (0.0)	NA^2^
D8	2/63 (3.2)	0/9 (0.0)	2/12 (16.7)	0/42 (0.0)	0.012
*C. guilliermondii*	3/355 (0.8)	1/55 (1.8)	1/46 (2.2)	1/254 (0.4)	0.331
D1	2/196 (1.0)	0/27 (0.0)	1/20 (5.0)	1/149 (0.7)	0.166
D5	0/96 (0.0)	0/19 (0.0)	0/14 (0.0)	0/63 (0.0)	NA^2^
D8	1/63 (1.6)	1/9 (11.1)	0/12 (0.0)	0/42 (0.0)	0.047
*C. orthopsilosis*	2/355 (0.6)	1/55 (1.8)	0/46 (0.0)	1/254 (0.4)	0.380
D1	2/196 (1.0)	1/27 (3.7)	0/20 (0.0)	1/149 (0.7)	0.315
D5	0/96 (0.0)	0/96 (0.0)	0/14 (0,0)	0/63 (0.0)	NA^2^
D8	0/63 (0.0)	0/8 (0.0)	0/11 (0.0)	0/39 (0.0)	NA^2^
*C. metapsilosis*	1/355 (0.3)	1/55 (1.8)	0/46 (0.0)	0/254 (0.0)	0.065
D1	0/196 (0.0)	0/27 (0.0)	0/20 (0.0)	0/149 (0.0)	NA^2^
D5	1/96 (1.0)	1/19 (5.3)	0/14 (0.0)	0/63 (0.0)	0.129
D8	0/63 (0.0)	0/8 (0.0)	0/11 (0.0)	0/39 (0.0)	NA^2^

^1^Data are presented as No. (%) unless otherwise specified. ^2^Not applicable.

If we particularize by hospital unit, 61 out of 101 isolates (60.4%) in the FFH ICUs were NAC species, compared with 109 out of 254 isolates (42.9%) in the BAH ICU. In terms of the distribution of *C. albicans* versus NAC species during the first week of hospitalization, there was a slight increase in *C. albicans* isolates across all ICUs, rising from 48.5% on day 1 to 55.2% on day 5, and 58.7% by day 8 (**Table 1**).

**Figure 2:**
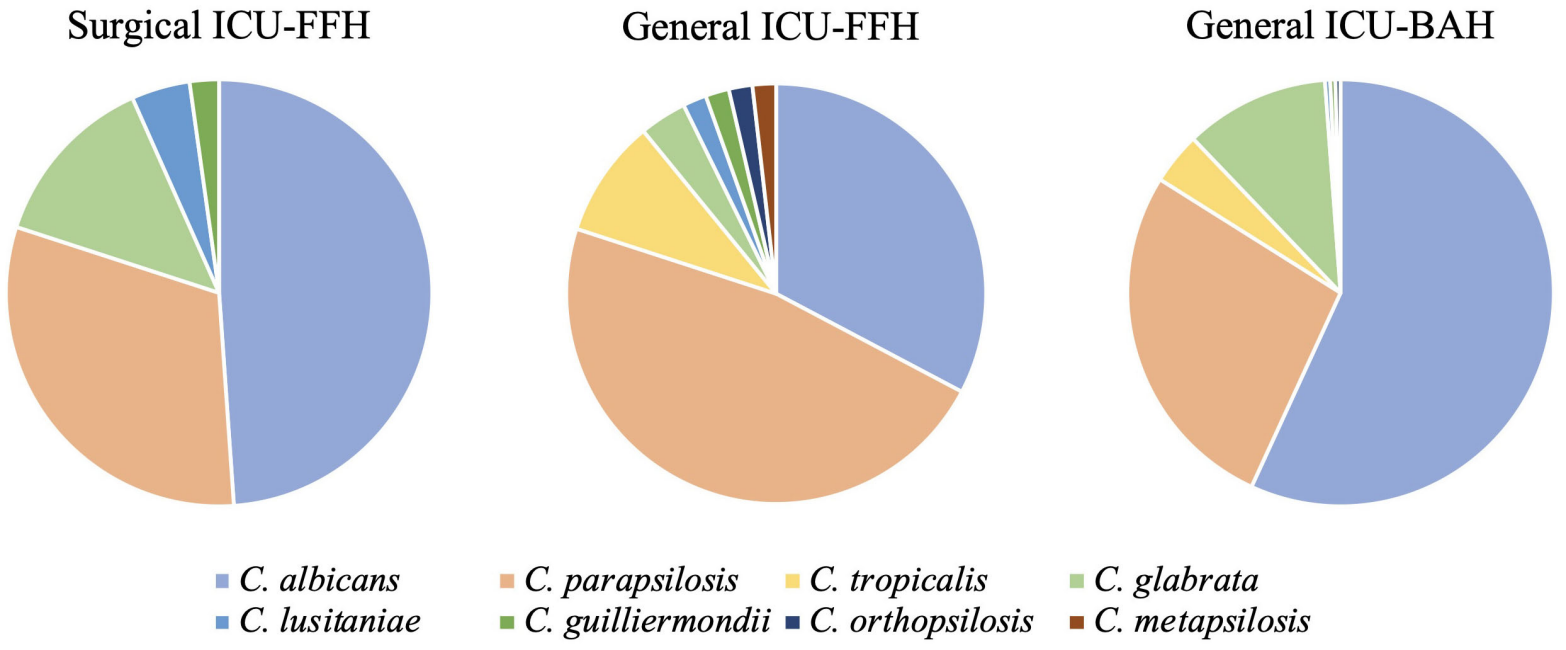
Distribution of *Candida* spp. isolates by ICU (general and surgical FFH and General BAH).

When analyzing the distribution of *Candida* species by ICU and hospital unit, species diversity was lower in the surgical FFH ICU. In contrast, both the general BAH and FFH ICUs exhibited the presence of all identified *Candida* species, although the percentage distribution of each species varied significantly (**Figure 2**).

The dynamics of *Candida* spp. colonization by ICU were significant at the three-point collections. Upon admission, *C. albicans* was associated with the BAH ICU (*p*=0.003). *C. parapsilosis* ranked second overall, except in the general ICU of the FFH, where it became the most prevalent species, representing 47.3% of isolates (*p*=0.011). Cryptic isolates from the *C. parapsilosis* complex were detected exclusively in the general ICUs of both the FFH and BAH, although this finding lacked statistical significance (**Table 1**). Interestingly, *C. tropicalis* was absent from all collection points in the surgical ICU of FFH. Despite the low number of *C. tropicalis* isolates overall, its distribution was significantly associated with the general ICU of FFH upon admission (*p*=0.006) (**Table 1**). Additionally, *C. parapsilosis* and *C. lusitaniae* were associated with specific collection points in the FFH ICUs. *C. parapsilosis* showed a significant association with the general ICU on day 5 (*p*=0.047), whereas *C. lusitaniae* was significantly linked to the surgical ICU on day 8 (*p*=0.012) (**Table 1**).

**Figure 3:**
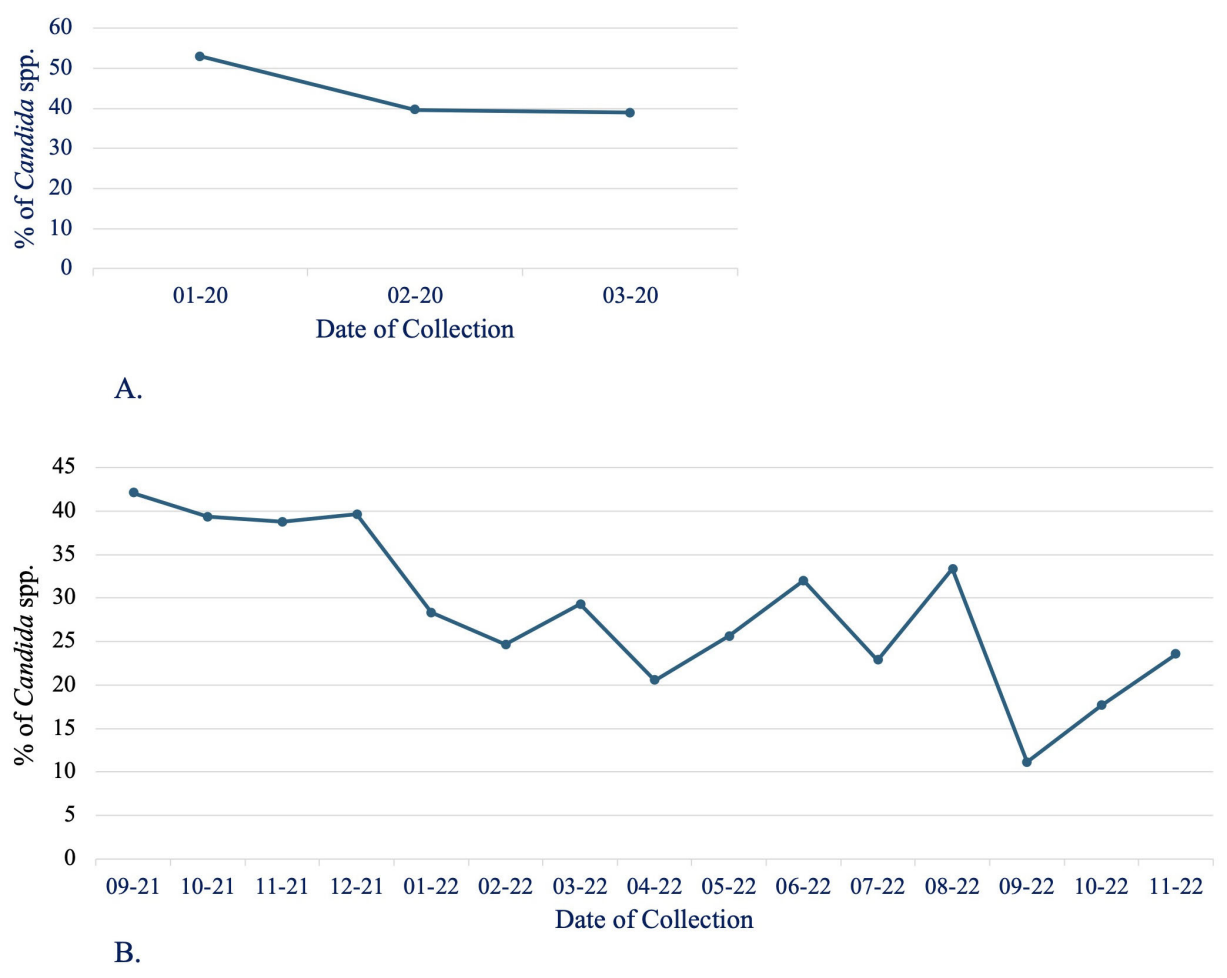
Distribution of *Candida* spp. isolates by month and hospital ICU. **(A)** General and surgical FFH and **(B)** General BAH.

Regarding the prevalence of *Candida* species, no significant month-to-month variations were observed during the collection period within the same hospital (**Figures 3A, B**). This suggests a consistent presence of *Candida* spp. throughout the year, without notable fluctuations in colonization rates across different months.

However, a broader analysis over a 3-year period revealed a noticeable decrease in the percentage of *Candida*-positive samples, dropping from 44.7% in 2020 to 40.2% in 2021, and further down to 25.6% in 2022. Although this downward trend was evident, it did not reach statistical significance, implying that although there may be a reduction in *Candida* prevalence, the change is not robust enough to rule out the possibility of random variation.

### 3.3 Specificity and sensitivity of the MSAA medium

**Figure 4:**
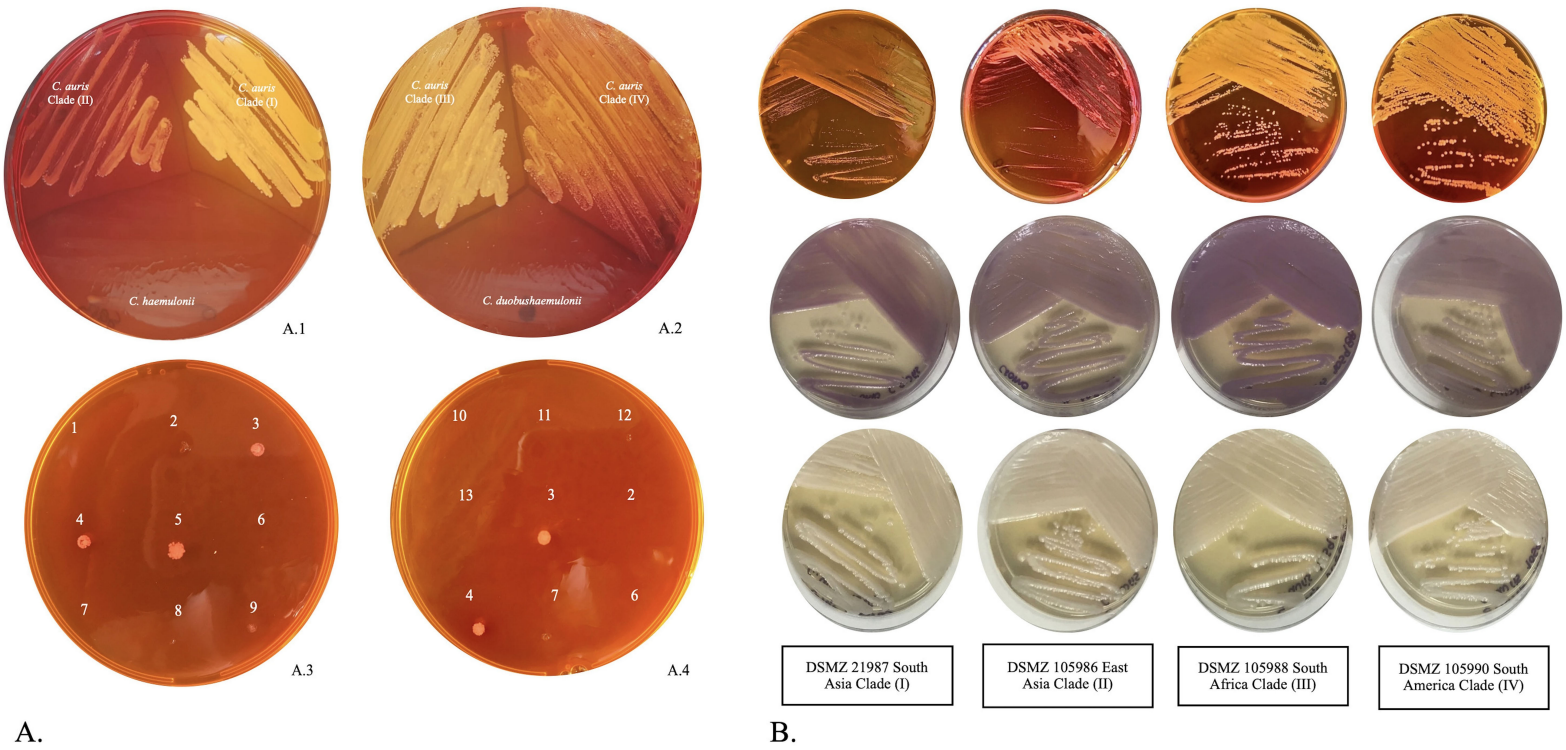
(**A**, A.1) MSAA plate inoculated with *C. auris* DSMZ 105986 (Clade II) and DSMZ 105987 (Clade I), and *Candida haemulonii* DSMZ 70624); (A.2) MSAA plate inoculated with *C. auris* DSMZ 105988 (Clade III) and DSMZ 105990 (Clade IV), and *Candida duobushaemulonii* CBS 7798. The MSAA plates were incubated for 48 h at 40°C; (A.3, A.4) MSAA sensitivity, growth at 0.1 McFarland. The *Candida* spp. tested were: 1 = *Candida albicans*; 2 = *Candida duobushaemulonii*; 3 = *C*. *auris* DSMZ 105990 (Clade IV); 4 = *C*. *auris* DSMZ 105987 (Clade I); 5 = *C*. *auris* DSMZ 105988 (Clade III); 6 = *Candida haemulonii* DSMZ 70624; 7 = *Candida glabrata*; 8 = *Candida parapsilosis*; 9 = *C*. *auris* DSMZ 105986 (Clade II); 10 = *Candida krusei*; 11= *Candida tropicalis*; 12 = *Trichosporon mucoides*; and 13 = *Saccharomyces cerevisiae*. **(B)** Macroscopic observation of *C*. *auris* clades I, II, III, and IV after 48 h of incubation at 37°C. MSAA plates inoculated by streaking from suspensions with 10^6^ CFU/ml of the control strains (top row). In CHROMagar Candida, *C*. *auris* cultures usually develop an appearance of various shades of pink (center row). On Sabouraud Dextrose Agar (SDA), the colonies generally remain white and creamy (bottom row).

Regarding the evaluation of the MSAA medium for the selective and differential isolation of *C. auris* control strains, after 48 h of incubation at 40°C, small yellow colonies were observed for the strains representing the major phylogeographic clades of *C. auris* (Clade I, II, III, and IV), whereas none of the remaining yeast species could grow on the same medium (**Figure 4A**). It is worth mentioning that *C. auris* DSM 105986 (East Asia: Japan, clade II) was the slowest growing strain. All strains grew on SDA plates and CHROMagar *Candida*, evidenced by the formation of white to cream and pink colonies, respectively, assuring the viability of all cultures (**Figure 4B**). Phylogenetically related species, such as *C. haemulonii* and *C. duobushaemulonii*, did not grow on the MSAA medium (**Figures 4**A.1, A.2). Similarly, the remaining 10 reference strains of other yeast species showed no growth on this selective medium at 40°C during the same incubation period.

Regarding sensitivity, the examination of MSAA plates with grid inoculation revealed consistent results even at low dilutions (standard suspension of 0.1 McFarland). For various *Candida* species, as well as *S. cerevisiae* and *T. mucoides*, MSAA revealed no growth for any of the isolates after 24 h of incubation, including *C. auris*. However, after 48 h of incubation, strains representing the geographical clades of *C. auris* exhibited growth, with the appearance of yellow colonies [**Figures 4**A.3, A.4, (compare inoculation points 1–13)]. The SDA plates used as controls to verify the viability of the isolates displayed creamy white colonies for all the reference strains, thereby validating the reliability of the MSAA medium results (**Figure 4B**). Based on these findings, the sensitivity of the MSAA medium for the presumptive detection of *C. auris* was determined after a 48-h incubation period, yielding a sensitivity of 100%.

### 3.4 The detection of presumptive *C. auris* isolates with culture-based methods

SSDB and MSAA media showed growth after inoculation with 51 and 21 surveillance swab samples, respectively. All MSAA-positive plates also evidenced turbidity in the enrichment broth. However, the SSDB yielded positive results for an additional 30 samples (**Table 2**).

**Table 2:** Presumptive identification results obtained with culture media (SSDB and MSAA) used to screen for *C. auris* vs. definitive identification.

Definitive identification^1^	No. of isolates	Collection isolates	Surveillance isolates	Positive results of inoculation	
				SSDB	MSAA
*Candida albicans*	186	1	185	5	0
*Candida parapsilosis*	110	1	109	32	15
*Candida glabrata* ss	37	1	36	8	1
*Candida tropicalis*	16	0	15	2	2
*Candida lusitaniae*	4	0	4	2	2
*Candida guilliermondii*	3	0	3	1	0
*Candida krusei*	1	1	0	0	0
*Candida orthopsilosis*	2	0	2	1	1
*Candida metapsilosis*	1	0	1	0	0
*Candida haemulonii*	1	1	0	0	0
*Candida duobushaemulonii*	1	1	0	0	0
*Candida auris* (Clade I)	1	1	0	1	1
*Candida auris* (Clade II)	1	1	0	1	1
*Candida auris* (Clade III)	1	1	0	1	1
*Candida auris* (Clade IV)	1	1	0	1	1
*Trichosporon* spp.	6	1	5	0	0
*Saccharomyces cerevisiae*	1	0	1	0	0
Total	373	12	361	55^2^	25^2^

^1^Definitive identification by MALDI-TOF and DNA-based methods. ^2^Total positive results including collection isolates.

The isolates retrieved from these two media were identified by MALDI-TOF MS as *C. glabrata*, *C. tropicalis*, *C. lusitaniae*, *C. guilliermondii*, *C. parapsilosis*, and *C. orthopsilosis*. The definitive identification of the isolates of *C. glabrata* and *C. parapsilosis* complexes was obtained using multiplex PCR. This identification was identical to that obtained from the primary isolation media used in the fungal colonization screening protocol. However, 16 samples were considered positive only after being cultured in the enrichment broth. Among the isolates from these samples, two of *C. lusitaniae*, five of *C. glabrata*, and nine of *C. parapsilosis* were retrieved. From these 16 samples, after transferring from dulcitol broth to CHROMagar Candida and MSAA, growth was observed only in MSAA for two samples, which were identified as *C. lusitaniae*.

The results of the cultural and molecular examination of combined axillary/inguinal mucosa swabs are summarized in **Table 2**. No sample with growth on either MSAA or SSDB was confirmed as positive for *C. auris* by MALDI-TOF or PCR. Based on these results, the specificities of SSDB and MSAA culture alone for the presumptive detection of *C. auris* from swab samples were 88.5% and 94.8%, respectively.

## 4 Discussion

Our results highlight the variability of *Candida* species frequency depending on the local hospital epidemiology, and our study evidenced the importance of the ICU type (surgery vs. general), as already mentioned by other authors (Guinea et al., 2014). Findings from this work are in line with previous studies (carriage/infection) worldwide, showing that *C. albicans* still ranks as the main yeast colonizer on ICU patients but, in the last decade, a change in favor of NAC has been observed in the ICU (Sasso et al., 2017; Boattini et al., 2023).

Sequential sampling to assess the dynamics of *Candida* species over time revealed stable results, with a tendency toward the prevalence of *C. albicans* over NAC species. This stability in colonization patterns aligns with previous longitudinal studies that used respiratory tract samples (Willger et al., 2014; Krause et al., 2016). Our results demonstrate that *C. albicans* consistently maintained colonization, reinforcing the established understanding of its virulence factors. *C. albicans* is known to possess the most extensive range of virulence traits among *Candida* species, enabling it to persist and maintain long-term viability within the host (Staniszewska, 2020). In contrast, NAC species were more often associated with transient colonization, with species turnover observed between ICU admission and collection after 1 week of hospitalization (day 8).

The observed decline in *Candida* spp. prevalence between 2020 and 2022 could be attributed to several factors, including improvements in infection control practices, shifts in patient demographics, or the impact of external influences such as the COVID-19 pandemic, which altered hospital admission patterns and antimicrobial usage. However, the lack of statistical significance in this trend indicates that further data collection and extended observation periods are needed to determine whether this decline is truly meaningful or simply part of natural variability. Understanding these trends is essential for guiding future surveillance efforts, optimizing infection control strategies, and anticipating potential changes in *Candida* prevalence within hospital settings.

The *Candida parapsilosis* complex was prevalent (31.5%) among NAC species, in agreement with previous studies that showed *C. parapsilosis* as the second most common species after *C. albicans* found at Southern European hospitals (Portugal, Spain, Italy, and Greece) (Castanheira et al., 2020; Boattini et al., 2023). It is worth noting that *C. parapsilosis* ranked first at admission for one ICU (*p*=0.011). This cohort of patients was enrolled in our study before the COVID-19 pandemic, and we may point to the reinforcement of infection control measures taken during the pandemic years, as the hands of healthcare professionals are recognized as a major vector for *C. parapsilosis* nosocomial acquisition.

Of the 36 isolates identified as *C. glabrata*, all strains were confirmed to be *C. glabrata* stricto sensu. These findings align with other studies that have investigated the presence of cryptic species within the *glabrata* complex and found no evidence of their existence (Kaan et al., 2021; Nasri et al., 2023). Only cryptic species of the *C. parapsilosis* complex were isolated. These results point to a stability in the distribution and emergence of cryptic species in the ICU setting in Portugal (Faria-Ramos et al., 2014).

*C. tropicalis* is typically derived from the gastrointestinal tract, which may account for its low prevalence of only 4.2% among the *Candida* isolates. Additionally, *C. lusitaniae* was identified in the cohort at a frequency of 1.1%, with a significant association found in the FFH surgical ICU (*p*=0.012). This pattern reflects similar findings reported in Portugal, where *C. lusitaniae* was present at a prevalence of 2.6% (Pinto-Magalhães et al., 2019).

Our screening to unveil *C. auris* in ICU patients relied on qPCR given the critical need for rapid identification to implement public health measures promptly. Nonetheless, MSAA medium showed a specificity of 94.8%, albeit *C. auris* was not detected in this cohort. Commercial culture media used to differentiate *C. auris* from other *Candida* species also presented misidentification issues. Although CHROMagar Candida Plus *C. auris* can identify all *C. auris* strains, other species were misidentified as *C. auris*, such as *C. parapsilosis* complex species (Taverna et al., 2023). Additional selective media were described for the isolation of *C. auris* (Ibrahim et al., 2021; Das et al., 2021). As for MSAA, these selective media are based on the yeast growth conditions: thermo-resistance and halo-tolerance (Jeffery-Smith et al., 2018). MSAA has less inhibitors (as we used a 40°C incubation temperature instead of 42°C and 10% of sodium chloride instead of 12.5%) and stable conservation (4°C) after medium preparation compared with medium described by Das et al., which requires fresh preparation (Das et al., 2021). All *C. auris* strains representing different major clades were able to grow on MSAA plates. However, as for the study by Das et al., the *C. auris* clade II strain showed limited growth even after 48 h of incubation time, due to its longer doubling time at 42°C (Das et al., 2021). Over the SCA described by Ibrahim et al., MSAA has the advantage of reading at 48 h instead of 72 h (Ibrahim et al., 2021).

In our study, MALDI-TOF MS-based identifications showed total agreement with molecular procedures, reinforcing other authors who stated that MALDI-TOF MS systems can correctly identify *C. auris* in most cases (Mahmoudi et al., 2019). Overall, our results show that when qPCR is not available, the MSAA medium can be used for the routine screening of *C. auris*. The MSAA medium can be easily prepared, is straightforward to use as it does not require mycological or molecular expertise, and provides a simple and cost-effective tool for the detection and presumptive identification of *C. auris* in the clinical microbiology laboratory. From these findings, the proposed workflow to identify *C. auris* based on culture-based methodologies associated with the molecular identification of the isolates is a good option and allows antifungal susceptibility tests to be performed.

To the best of our knowledge, this prospective study is the first surveillance study for *C. auris* colonization in Portuguese tertiary hospital ICUs. One major strength is that it is specifically designed to screen for *C. auris* in a cohort of patients from two large hospital centers in the suburban Lisbon region. For over 2 years, our findings showed that *C. auris* is not prevalent in Portuguese hospitals. Our findings align with other prospective screening studies, namely from the UK (Sharp et al., 2021), Egypt (Khairat et al., 2021), and Germany (Heindel et al., 2022), which also reported no *C. auris* colonization among ICU patients. Additionally, our results are consistent with observations from Europe and North America, where *C. auris* colonization and infection in ICUs are typically sporadic and often associated with nosocomial outbreaks (Pacilli et al., 2020; Worth et al., 2020; Eckbo et al., 2021; Piatti et al., 2022). However, the situation is markedly different in India, where *C. auris* has become the leading *Candida* species isolated from the blood of critically ill patients, with a notable increase in cases during the COVID-19 pandemic (Shastri et al., 2020; Sharma and Chakrabarti, 2023).

In Europe, new *C. auris* cases were mainly diagnosed during the COVID-19 pandemic, particularly in Spain (n=591), Italy (n=291), and Greece (n=71) (Kohlenberg et al., 2022). Nevertheless, several studies carried out all over Europe in recent years reported only isolated cases of *C. auris* in hospital units, such as with the first Portuguese clinical case (Kohlenberg et al., 2022; Henriques et al., 2023). Furthermore, a multicentric retrospective study including all candidemia case isolates from six Southern European tertiary hospitals did not find *C. auris* (Boattini et al., 2023). In a 2022 prospective screening study by Contreras et al., *C. auris* isolates were recovered from critically ill patients with complicated clinical conditions who had a history of medical care not only in intensive care units but also in long-term care facilities (Contreras and Morgan, 2022). Similarly, the CDC’s pilot study on *C. auris* screening in several healthcare facilities across New York City between 2017 and 2019 found that colonizing isolates were predominantly obtained from patients in long-term care facilities and senior residences (Rowlands et al., 2023).

As the patients in these two hospitals did not remain in the ICU for extended periods (e.g., a median of 5 days), they may not have been exposed to the cumulative risks that typically lead to *C. auris* colonization. A shorter stay reduces the likelihood of encountering contaminated environments, decreases the time exposed to other colonized patients, and limits the use of invasive devices that could serve as entry points for *C. auris*. The absence of *C. auris* colonization in the two hospitals could indeed be due to the relatively short duration of hospital stays among the participants. This aligns with existing literature that indicates a higher risk of *C. auris* colonization after prolonged hospital or ICU stays, typically at approximately or after 20 days (Corcione et al., 2022). Das et al. (2018) highlighted a critical observation that patients in ICU settings typically acquired *C. auris* infections after a period of 15 to 20 days (Das et al., 2018).

Another potential limitation of our study was the collection of samples from only two sites for *C. auris* screening in the ICU setting. This approach may have reduced detection effectiveness, as Rowlands et al. (2023) emphasized the importance of including nasal swabs alongside bilateral axillary/inguinal mucosal sampling for comprehensive screening (Rowlands et al., 2023). Additionally, the study collected fewer swabs than anticipated (988 instead of 1,350) due to its premature termination. This early conclusion was influenced by several factors, including logistical challenges and external events, which resulted in fewer patients being swabbed than initially planned. Specifically, in the two ICUs at FFH, the study was halted prematurely in March 2020 due to the onset of the COVID-19 pandemic. The pandemic disrupted hospital research activities, leading to an early end to the study. At BAH, from October 2022 onwards, staff shortages created significant difficulties in continuing the study, particularly in the collection of swabs. These staffing limitations ultimately forced an early end to the research. Understanding the specific reasons behind these premature terminations is crucial for planning and designing future research studies.

Although *C. auris* colonization currently appears to be uncommon in Portuguese hospitals, Portugal has seen a significant increase in the number of immigrants from areas endemic for *C. auris*. This trend raises concerns about the possibility of the fungus being introduced and spread in the country and suggests that Portuguese hospitals should consider the protocol presented for an active screening.

Considering the above findings, this study, along with the various studies cited in the Discussion, underscores the need to implement *C. auris* screening alongside existing protocols for nosocomial microorganisms such as methicillin-resistant *Staphylococcus aureus*, vancomycin-resistant *Enterococcus*, and carbapenemase-producing Gram-negative bacteria. It is recommended that nasal and rectal swabs, in addition to axillary and inguinal swabs, be utilized for more comprehensive screening. Furthermore, screening should be systematically extended beyond the ICU to include palliative care units to enhance the early detection and management of *C. auris*.

In conclusion, by integrating molecular technologies with traditional culture assays, a screening protocol for *C. auris* was developed and implemented. This protocol involves initial screening using MSAA to identify potential *C. auris* isolates, followed by confirmation using traditional PCR or MALDI-TOF MS. Through our study, we also uncovered the colonization pattern of *Candida* in the ICU and contributed to the optimization of overall patient management.

## Data Availability

The original contributions presented in the study are included in the article/supplementary material. Further inquiries can be directed to the corresponding author.
